# BRIGHT-HD—A Brazilian Research Investigation on Public Health Gains Comparing Survival Rates Between Hemodialysis and Hemodiafiltration: An Observational Study

**DOI:** 10.3390/jcm14113981

**Published:** 2025-06-05

**Authors:** Eduardo P. Luciano, João Chang, Elaine C. S. Arantes, Aline Cordeiro, Sandra F. S. Reis, Douglas V. Andrade, Whelington F. Rocha, Andrea O. Magalhães, Cynthia M. Borges, Rosilene M. Elias

**Affiliations:** 1 Splendore Kidney Care, Sao Paulo 01438, Brazil; cynthianefro@gmail.com (C.M.B.); rosilenemotta@hotmail.com (R.M.E.); 2 Fundação Lia Maria Aguiar, Instituto de Responsabilidade Social Sírio Libanês, Campos do Jordão 12460, Brazil; aline.cordeiro@nefrostar.com.br (A.C.); sfstreis@uol.com.br (S.F.S.R.); douglas.vandrade@flmasaude.org.br (D.V.A.); 3 Hospital Regional do Vale do Paraíba, Taubaté 12030, Brazil; jchang@terra.com.br (J.C.); elaine.arantes@hospitalregional.org.br (E.C.S.A.); 4 Nefrostar Kidney Care Nefrostar, Sao Paulo 06010, Brazil; rochafiton@yahoo.com.br (W.F.R.); andrea.olivares@terra.com.br (A.O.M.); 5 Department of Medicine, Service of Nephrology, Hospital das Clínicas HCFMUSP, Sao Paulo 05403, Brazil; 6 Post-Graduation, Universidade Nove de Julho (UNINOVE), Sao Paulo 01504, Brazil

**Keywords:** arteriovenous fistula, chronic kidney disease, dialysis, mortality

## Abstract

**Background/Objectives:** High-flux online hemodiafiltration (OL-HDF) appears to be associated with better survival than hemodialysis (HD). In Brazil, OL-HDF is only affordable for patients with private health insurance. Although observational studies have shown a survival advantage with OL-HDF, even in Brazil, it is unclear whether this benefit applies to patients without private health insurance. We compared overall and cardiovascular mortality between OL-HDF and HD in patients treated exclusively through the public health care system. We hypothesized that patients on OL-HDF would have a higher survival rate than those on HD. **Methods:** This is an observational cohort study. Adult patients on maintenance hemodialysis or OL-HDF for at least one month during the period between 1 September 2022 and 1 December 2024 were enrolled into the study. The primary outcome was all-cause mortality. The secondary outcome was cardiovascular mortality. Fine-Gray sub-distribution hazard models were used to evaluate survival in the presence of competing events (kidney transplant and recovery of renal function). **Results:** Patients on HD (N = 321) and OL-HDF (N = 48) were similar in age, race, sex, and vascular access. Patients on HD were more likely to have diabetes (54.0% vs. 29.2%, *p* = 0.001) and spent more hours per week on dialysis (11.2 ± 1.8 vs. 10.5 ± 1.6 h, *p* = 0.006). In an adjusted Fine-Gray model, the hazard of death for patients on OL-HDF was 68% lower than that for patients on HD, and the risk of death for patients with an arteriovenous fistula was 55% lower compared to those with a catheter. Cardiovascular mortality did not differ between the groups. **Conclusions**: These findings suggest that OL-HDF is associated with an overall higher survival rate compared to HD, even for patients without private health insurance.

## 1. Introduction

Hemodialysis (HD) is the most often performed modality of renal replacement therapy in Brazil [1] and around the world [2]. However, the mortality rate among patients on maintenance HD remains unacceptably high. Following the need for innovations, several studies have shown that hemodiafiltration (HDF), a therapy that combines diffusion and convection, has provided better survival outcomes compared to HD. Among these studies, a Turkish study [3], the ESHOL study [4], and the CONVINCE study [5] have demonstrated an improvement in survival rates, although some criticism still exists regarding some discrepancies between groups.

In Brazil, 80.3% of patients on hemodialysis are covered by the public health care system [6], and access to HDF is restricted to patients without private health insurance. When attempting to replicate survival comparisons between HD and HDF, bias is inevitable, as patients on HDF generally have better healthcare access and a higher overall socioeconomic status. Therefore, in Brazil, such a comparison is not fair, as it involves more than just a comparison of dialysis modalities. To date, only one study in Brazil has shown a survival advantage for HDF over HD, with a hazard ratio of 0.29 (0.11–0.77), in a propensity score analysis [7]. However, since the comparison was between patients with and without private health insurance, doubt remains regarding non-measured factors that might have influenced the results.

In the present study, we aimed to compare mortality between HD and online HDF patients without private health insurance. We hypothesized that patients on OL-HDF would have a higher survival rate than those on HD.

## 2. Materials and Methods

This is a retrospective cohort study comparing overall and cardiovascular mortality among patients receiving HD and online HDF (OL-HDF) in the absence of private health insurance.

### 2.1. Study Population

Patients on OL-HDF were recruited from the dialysis center at the Fundação Lia Maria Aguiar, a philanthropic dialysis center that provides HDF for all patients. Those on HD were recruited from a regional hospital in the public health care system. At both sites patients had no private health insurance.

Inclusion criteria were adult patients on maintenance dialysis for at least 1 month in the period between 1 September 2022, and 1 December 2024. Exclusion criteria were adult patients with acute kidney injury or those hospitalized in the last 3 months.

The sample size was obtained by convenience and all patients in both centers were screened. Patients were in follow-up at the point of entry into the study until the end of follow-up by reaching an endpoint or the end of the study.

Demographic, clinical, laboratory, and dialysis data were obtained from electronic charts and manually checked in each center. Data evaluated included age, sex, presence of diabetes, and type of vascular access (arteriovenous fistula or catheter) at the point of study entry. Data on dialysis prescriptions were also evaluated and included the frequency and number of dialysis hours per week.

For OL-HDF (Fresenius 5008 machine, Fresenius Medical Care, Bad Homburg, Germany) ultrapure dialysate and high-flux polysulfone membrane (Fresenius Cordiax Fx 1000, FX 800, and FX 600) were used. Except in some cases where there is a high risk of coagulation, post dilution OL-HDF is prescribed to achieve a substitution volume of at least 23 L per session. For HD (Fresenius 4008 machine), blood flow was set between 300–400 mL/min. The dialysate flow rate was 500 mL/min at both sites. Ultrafiltration was prescribed according to dry weight and physicians were free to adjust upon clinical evaluation. The potassium dialysate concentration was 2 mEq/L and the calcium was 2.5 or 3 mEq/L, with bicarbonate individualized around 30–32 mEq/L.

The study was conducted according to the Declaration of Helsinki. The local ethics committee approved the protocol (#84252424.0.0000.5511), and all patients provided written consent via a form. Clinical data were anonymized for safety and privacy.

### 2.2. Study Outcomes

The primary outcome was all-cause mortality. All deaths and causes of deaths observed during the study period were recorded. The secondary outcome was cardiovascular mortality, defined as stroke, myocardial infarction, or heart failure as the primary cause of death.

All patients were followed until death, a kidney transplant, the recovery of renal function, the loss of follow-up, or the end of the study period.

### 2.3. Statistical Analysis

Continuous variables were presented as mean ± standard deviation or median (25, 75 percentile) according to data distribution, which was checked using the Shapiro–Wilk test. Categorical data was presented as the number and percentage. Comparison between OL-HDF and HD groups was performed using the *t*-test or the Mann–Whitney U test for normally and not-normally distributed data, respectively. For categorical data, we used the chi-square or Fisher, as appropriate.

Fine-gray sub-distribution hazard models were used to evaluate survival in the presence of a competing event (e.g., a kidney transplant or the recovery of renal failure). In the initial multivariate model, all predictor variables were considered. Then, the non-significant variables were excluded one by one in order of significance (using the backward method). Interactions between the predictor variables and time were included in the final model—the non-significance of these variables suggests no violation of this assumption. For all statistical tests, a significance level of 5% was used. The analyses were performed using the statistical package STATA 17.

## 3. Results

The baseline characteristics are illustrated in Table 1. Age, race, and sex were similar between HD (N = 321) and OL-HDF groups (N = 58). Diabetes was more prevalent in patients on HD. More frequent dialysis (four or more times a week) was performed mostly among patients on OL-HDF, although this group had a lower number of hours on dialysis per week.

**Figure 1:**
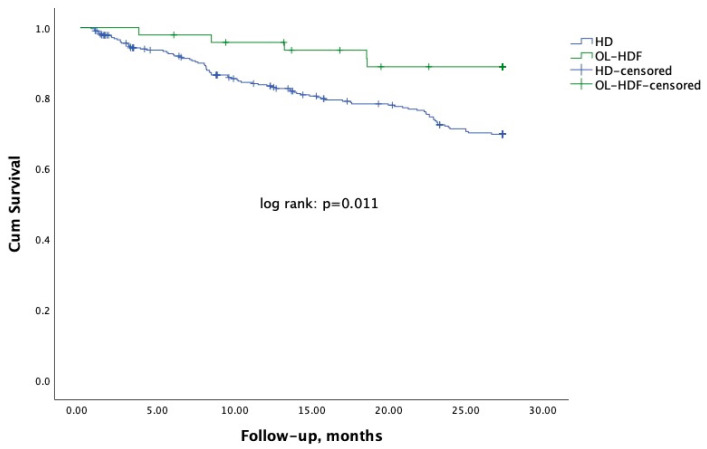
Kaplan Meier survival analysis according to dialysis modality, online hemodiafiltration (OL-HDF, green line), or hemodialysis (HD, blue line).

During a median follow-up time of 27.4 months, there were 92 deaths (87 on HD and 5 on OL-HDF). Recovery renal function (10 and 4 patients on HD and OL-HDF, respectively) and kidney transplantation (16 and 1 patients on HD and OL-HDF, respectively), were treated as competitive risks. Non-adjusted Kaplan Meier survival analysis (Figure 1) showed an advantage of mortality in patients on OL-HDF (log rank test *p* = 0.011).

**Figure 2:**
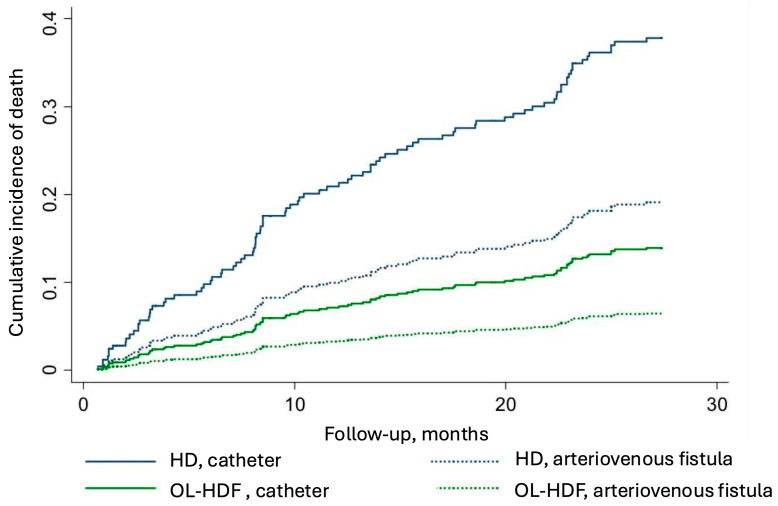
Cumulative number of events according to dialysis modality (online hemodiafiltration—OL-HDF or hemodialysis—HD) and type of vascular access (catheter or arteriovenous fistula). From the top to the borrow, lines represent patients on HD with a catheter (continuous blue line), patients on HD with arteriovenous fistula (dashed blue line), patients on OL-HDF with a catheter (continuous green line), and patients on OL-HDF with an arteriovenous fistula (dashed green line).

As shown in Table 1, the modality of dialysis (*p* = 0.015) and the presence of an arteriovenous fistula (*p* < 0.001) were significantly associated with a better overall survival in the univariate model. In the final adjusted model, the hazard of death for patients on OL-HDF was 68% lower than that for patients on HD. Additionally, the risk of death in patients with an AVF was 55% lower compared to patients with a catheter. Figure 2 illustrates the cumulative number of events according to dialysis modality and vascular access.

For cardiovascular mortality, we identified only seven events, and OL-HDF was not associated with better survival [HR: 2.59 (0.51–13.27), *p* = 0.254] in a model adjusted for age, diabetes, vascular access, and time on dialysis per week (Table 2).

## 4. Discussions

In this study, we found that OL-HDF was associated with a higher survival rate over a 2-year follow-up period. Even adjusting for the main factors influencing mortality in patients on maintenance dialysis, such as the presence of diabetes and the type of vascular access, OL-HDF showed a 68% lower incidence of all-cause mortality compared to patients on standard HD. Our study is unique because we included patients without private health insurance, which reduces the bias of selecting patients who have better overall access to healthcare.

The population included in this study shares some characteristics with the dialysis population in Brazil. Compared to data from the Census of the Brazilian Society of Nephrology [1], patients in the HD group have a higher percentage of diabetes (54.5% vs. 32%), a similar percentage of patients undergoing dialysis 4 or more times per week (5.9% vs. 1.1%), and a lower percentage of patients with an arteriovenous fistula (47.4% vs. 70.8%). The age of our patients is also comparable to that of a large cohort of individuals starting hemodialysis in many regions in Brazil [8]. HDF was performed in 22% of patients with private health insurance in 2022 [1], a modality that has been increasing despite the lack of formal guidelines from the National Supplementary Health Agency. Recently, a suggestion for prescribing HDF was published in a Brazilian journal [9], following the initiative on the implementation of the modality with the HDFit trial, a randomized study assessing the benefits of OL-HDF over HD, particularly in improving patients’ physical activity. [10]

The median follow-up in the current study was 27 months, which is similar to the follow-up period in the Turkish study [3] but shorter than the follow-up achieved in the CONVINCE study [5] (30 months) and the ESHOL study [4] (36 months). Nevertheless, despite the relatively short follow-up, we observed 92 cases of death, which provided enough events to strengthen the statistical analysis. However, for the cardiovascular mortality, the number of events was too small to allow for meaningful analysis.

Comparing mortality between patients with and without private health insurance in Brazil is challenging. A previous study found no difference in 1-year mortality rates among adult patients starting HD from January 2011 to December 2021 [11]. In contrast, another study involving adults undergoing HD between 2012 and 2017 across 21 dialysis centers in Brazil showed an increased risk of death for patients whose treatment was funded by the public health care system [12]. For incident patients, a population with a higher mortality risk, it seems that patients with private insurance have an advantage in survival [13]. Discrepancies among the results may be explained by differences in the populations included or in the statistical methods used, as Cox regression does not account for competing risks such as kidney transplant or recovery of renal function. Nonetheless, it is undeniable that patients who can afford private insurance are more likely to receive better overall care beyond just dialysis, including access to medical exams, treatment from other specialties, and hospitalization when needed.

Although Cox regression has been used long before the recognition of competing risks, it is now considered the most appropriate approach to assess survival in dialysis patients. In this population, follow-up for clinical events of interest is complicated by the possibility that a completely different event may occur first, such as a recovery of kidney function or a kidney transplant—which could prevent the occurrence of the event of interest [14]. Indeed, a previous study in patients with chronic kidney disease (CKD) showed that traditional methods increasingly overestimated the risk of an event (such as kidney failure) as the follow-up time lengthened [15].

Recently, our group compared patients on HD and HDF in Brazil in a propensity score matched study 2:1 (i.e., 170 patients on public health HD: 85 patients on private insurance OL-HDF) [7]. The authors showed that HDF was associated with a reduced risk of mortality, after adjusting for age, type of access, Kt/V, hemoglobin, and phosphorus. Even with multiple adjustments, the question of whether HDF is superior remains influenced by the additional care that patients with private insurance receive. Moreover, private dialysis facilities in Brazil tend to offer more intensive multidisciplinary care, which includes physiotherapy, a factor that could impact the results beyond the dialysis modality itself. To that end, the current study is less likely to be biased, as the only difference among the dialysis centers was the dialysis modality. However, we cannot completely rule out the potential contribution of ultrapure water treatment, a condition that was only available to patients on HDF, and has been associated with lower inflammation [16] and mortality [17].

The presence of an arteriovenous fistula was associated with a lower risk of death in our study, which aligns with existing literature showing a significant impact on cardiovascular [18] and all-cause mortality [19]. This benefit is still controversial in older patients [20]. Overall, fistula remains the preferred choice for vascular access in hemodialysis [21]. Longevity on dialysis is proportional to the quality of the treatment, which in turn depends on access, ensuring an adequate flow rate to deliver the dialysis prescription. We demonstrated that the arteriovenous fistula was associated with a lower risk of death in patients on hemodialysis, a benefit that was further enhanced when OL-HDF was incorporated.

Our study should be evaluated considering the following limitations: 1. We do not have data on nutritional markers, phosphorus, and albumin; 2. The vascular access as a reference was the initial in use at the time of study inclusion; 3. There was an imbalance between the groups, particularly in the prevalence of diabetes. However, we found that the hazard ratio for the modality did not change in the multivariate models, indicating that diabetes did not significantly influence our results. 4. We included prevalent dialysis patients, so mortality related to the initiation of dialysis could not be assessed. 5. The study design did not allow for the establishment of a cause–effect relationship. Our strengths include, firstly, our use of a competing risk analysis, which is more appropriate for the dialysis population. Secondly, we are the first in Brazil to compare HD and HDF in patients without private health insurance.

We conclude that OL-HDF was associated with a lower risk of death over a 2-year follow-up in a population without private health insurance in Brazil.

## Figures and Tables

**Table 1:** Characteristics of patients according to group hemodialysis (HD) or online hemodiafiltration (OL-HDF).

Variable	HD N = 321	OL-HDF N = 48	*p*
Age, years	60 ± 14	61 ± 14	0.676
White race, n (%)	212 (66.0)	32 (66.7)	0.932
Men, n (%)	176 (54.8)	30 (62.5)	0.318
Arteriovenous fistula, n (%)	152 (47.4)	21 (43.8)	0.641
Diabetes, n (%)	175 (54.5)	14 (29.2)	0.001
Dialysis frequency/week, %			<0.001
Three times	94.1	66.7	
Four or more times	5.9	33.3	
Dialysis vintage, months	75.1 (33.4, 156.0)	35.3 (6.2, 68.2)	<0.001
Week dialysis duration, h	11.2 ± 1.8	10.5 ± 1.6	0.006

Unless otherwise specified. Data are expressed as mean ± SD or median (25, 75).

**Table 2:** Fine and Gray model for all-cause mortality.

	Univariate Model		Initial Multivariate Model		Final Multivariate Model	
	Crude HR (IC 95%)	*p*	Adjusted HR (IC 95%)	*p*	Adjusted HR (IC 95%)	*p*
HD—reference	0.33 (0.13–0.81)	0.015	0.32 (0.13–0.81)	0.015	0.32 (0.13–0.77)	0.011
Age, years	1.01 (1.00–1.03)	0.104	1.01 (0.99–1.03)	0.234	-	-
Diabetes	1.47 (0.97–2.23)	0.068	1.36 (0.88–2.09)	0.167	-	-
Arteriovenous fistula	0.46 (0.29–0.71)	<0.001	0.46 (0.29–0.72)	0.001	0.45 (0.29–0.69)	<0.001
Hours on dialysis/week	0.95 (0.87–1.05)	0.339	0.93 (0.85–1.03)	0.177		

HR, hazard ratio. N = 92, 31 and 146 cases of death, competitive events and censored cases, respectively. Final model proportional risk test (*p* = 0.992).

## Data Availability

Data supporting this research is available at https://data.mendeley.com/drafts/kmxhp4p5zr, accessed on 20 May 2025.
